# Dialectics of perisomatic inhibition—The unity and conflict of opposites

**DOI:** 10.3389/fncir.2024.1494300

**Published:** 2024-10-29

**Authors:** Andrei Rozov, David John Jappy, Ksenia Maltseva, Alina Vazetdinova, Fliza Valiullina-Rakhmatullina

**Affiliations:** ^1^Laboratory of Electrophysiology, Federal Center of Brain Research and Neurotechnologies, Moscow, Russia; ^2^Laboratory of Neurobiology, Kazan Federal University, Kazan, Russia; ^3^Institute of Neuroscience, Lobachevsky State University of Nizhniy Novgorod, Nizhny Novgorod, Russia

**Keywords:** perisomatic inhibition, oscillations, stress, GABA, network

## Abstract

Over the past three decades, a great deal of attention has been paid to the study of perisomatic inhibition and perisomatic inhibitory basket cells. A growing body of experimental evidence points to the leading role of perisomatic inhibitory cells in the generation of oscillatory activity in various frequency ranges. Recently the link between the activity of basket cells and complex behavior has been demonstrated in several laboratories. However, all this is true only for one type of perisomatic inhibitory interneuron—parvalbumin-positive basket cells. Nevertheless, where parvalbumin-positive basket cells are found, there is another type of basket cell, cholecystokinin-positive interneurons. These two types of interneurons share a number of common features: they innervate the same compartments of target neurons and they often receive excitation from the same sources, but they also differ from each other in the synchrony of their GABA release and expression of receptors. The functional role of cholecystokinin-positive basket cells in oscillatory activity is not so obvious. They were thought to be involved in theta oscillations, however recent measurements in free moving animals have put some doubts on this hypothesis. Therefore, an important question is, whether these two types of basket cells work synergistically or perform opposing actions in functional networks? In this mini-review, we attempt to answer this question by putting forward the idea that these two types of basket cells are functionally united as two entities of the same network, and their opposing actions are necessary to maintain rhythmogenesis in a “healthy”, physiological range.

## Introduction

Brain rhythms, i.e., fluctuating waves of neuronal activity that are observed using local field potential recordings, reflect the synchronized activity of large neuronal populations. Neuronal network oscillations provide a common timeframe for neuronal coupling both within local networks and between distant brain regions. Network oscillatory patterns strongly depend on vigilance and behavioral state (Buzsáki, 2002; Whishaw and Vanderwolf, 1973) and are thought to play a key role in memory formation by synchronizing the activity of distributed neurons and providing integrated representations of experiences during memory retrieval (Colgin, 2013; Draguhn et al., 2014).

It is generally accepted that interneurons play a vital role in the synchronization of downstream populations of excitatory neurons and, therefore, they can be considered as key elements in the generation of rhythmic brain activity. Over the past three decades of study of neural circuits and networks, much attention has been paid to exploring the functional role of perisomatic inhibition. Indeed, among the huge variety of different types of GABAergic interneurons, only perisomatic inhibitory basket cells and axo-axonal neurons are considered as distinct subpopulations that directly control the spike generation of the target cell. For example, numerous studies have shown the involvement of fast-spiking PV-positive basket cells (PVBCs) in fast hippocampal network oscillations such as sharp wave ripples (SPW-R) and gamma oscillations. Involvement of PVBCs in rhythmogenesis has been experimentally confirmed both in acute brain slices, and *in vivo* (Freund and Buzsáki, 1996; Klausberger and Somogyi, 2008; Buzsáki and Wang, 2012). Experimental evidence for the critical role of PVBCs in gamma oscillations and SPW-Rs has led to numerous computational models suggesting that these interneurons are the primary rhythm generators in the CNS (Börgers et al., 2005; Tiesinga and Sejnowski, 2009; Volman et al., 2011). The choice of PVBCs as the “usual suspect” responsible for network synchronization is based on the perisomatic nature of the inhibition provided by these interneurons. However, there is another type of GABAergic basket cell, cholecystokinin-positive basket cells (CCKBC), which have a similar pattern of synaptic integration to PVBCs, at least in the hippocampus. Both types of interneurons receive excitatory drive from the same sources and provide perisomatic inhibition of the same targets (Freund and Katona, 2007). However, although there is consensus that PVCBs are a key element in fast rhythm generation, the primary functional role of CCKBCs has not yet been determined.

In this mini-review we discuss recent findings that may help to elucidate the possible function of CCKBCs.

## Do PVBCs suppress or promote excitation flow?

The pace making ability of PVBCs arises from the following features of these interneurons:

(i) High initial probability and fidelity of GABA release at synapses formed by these cells.
(ii) Extensive gap-junction coupling that promotes synchronized activity of the PVBC population.
(ii) A high percentage of connectivity for both incoming excitatory inputs and outgoing inhibitory afferents.
(iv) The perisomatic nature of PVBC inhibition.

Obviously, there are other properties of PVBCs involved in the generation of oscillations, such as their ability to sustain high-frequency firing, and the expression of M1 muscarinic acetylcholine receptors (Yi et al., 2014), however, the four network and synaptic properties mentioned above are sufficient to assign PVBCs the role of the main suppressor of excitation.

**Figure 1:**
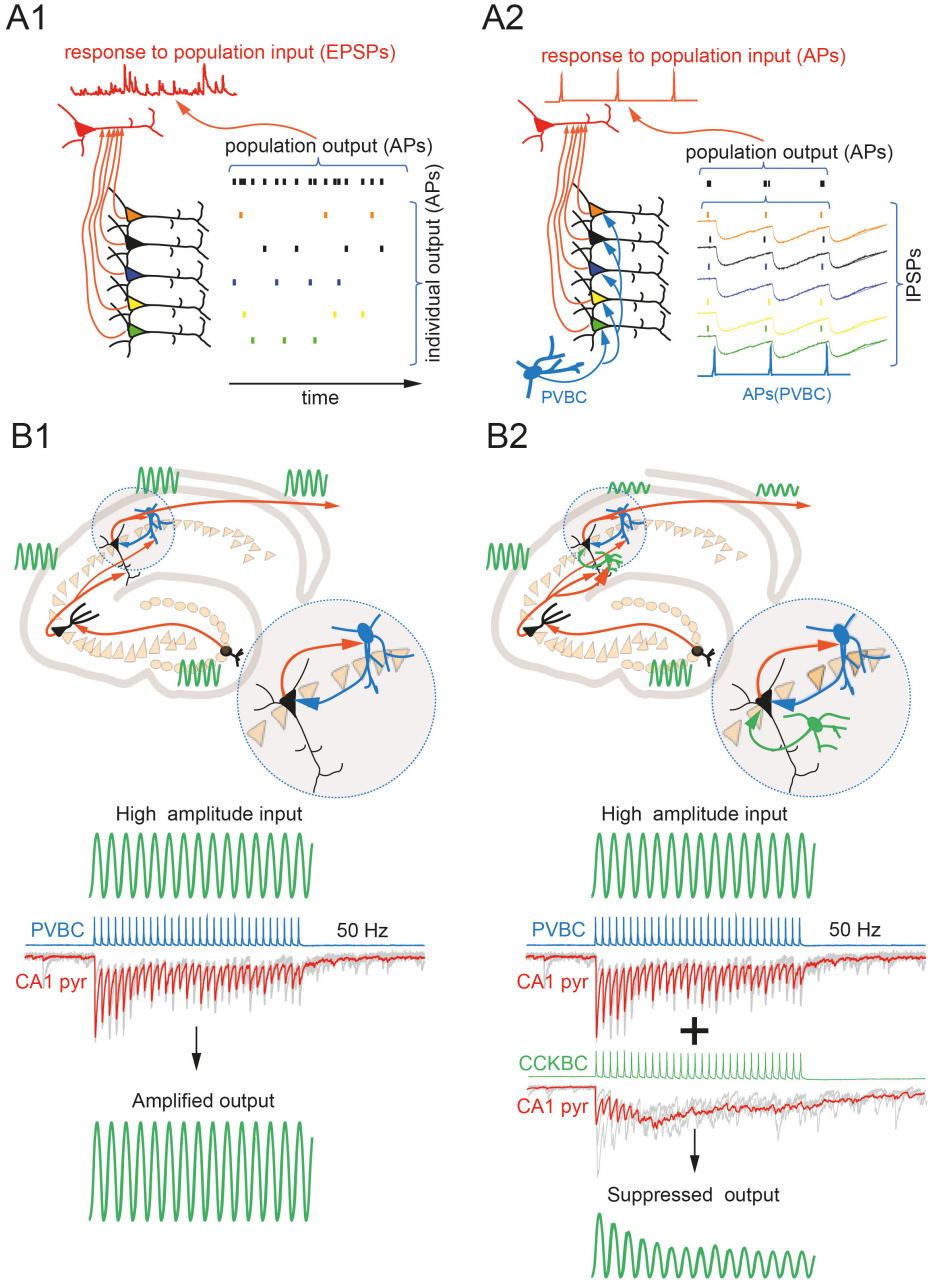
**(A)** PVBC-mediated perisomatic inhibition enhances the efficiency of excitation flow. In the absence of perisomatic inhibition, desynchronized population input results in a subthreshold EPSP burst in the target neuron **(A1)**. PVBC-generated perisomatic IPSPs narrow the AP-generation windows in the projecting neuronal population, resulting in efficient temporal summation of EPSPs to suprathreshold levels in the target cell **(A2)**. **(B)** CCKBC-mediated perisomatic inhibition compromises the potentiating effect of PVBCs on excitation during high-frequency rhythms. The schematic drawing illustrates the possible function of PVBCs as the booster of the rhythmic activity via the mechanism shown on **(A2)**. Gray traces are individual responses in a CA1 pyramidal cell to 50 Hz stimulation of PVBC. The red trace is the average of ten subsequent sweeps **(B1)**. Engagement of CCKBC in high-frequency rhythmic activity results in a marked asynchronous release of GABA, resulting in desynchronization of target neurons with the main rhythm. Gray traces are individual responses in a CA1 pyramidal cell to 50 Hz stimulation of CCKBC. The red trace is the average of ten subsequent sweeps **(B2)**.

While PVBCs do suppress excitation, population activity of PVBCs, does not necessarily lead to a decrease in the flow of excitation within the brain, since they may work to synchronize neuronal populations. In the central nervous system, EPSP amplitudes in most unitary connections are very small and insufficient to trigger an action potential in postsynaptic neurons (Feldmeyer and Sakmann, 2000). Low synaptic efficacy is more prominent at glutamatergic synapses between excitatory neurons (Koester and Johnston, 2005). Therefore, to reach the firing threshold, a neuron must simultaneously receive an excitatory signal from several presynaptic cells, which requires synchronization of projecting neurons. PVBCs are likely the most suitable candidates for synchronizing large populations of excitatory neurons (Figures 1A1, A2). Indeed, in the CA1 region of the hippocampus, a single PVBC can innervate more than 1,000 pyramidal neurons, the cell bodies of which are distributed across 1,000 μm in *stratum pyramidale* (Buhl et al., 1994; Halasy et al., 1996; Sik et al., 1995). Although in CA1 the density of PV-positive boutons is higher on deep pyramidal cell soma (Lee et al., 2014), during rhythmic activity all pyramidal cells receive massive PVBC-mediated inhibition. So, let's return to the question of the influence of PVBCs on the propagation of excitation between neuronal ensembles. The effect of inhibition provided by PVBCs is much stronger during oscillations such as sharp wave-ripples (SPW-R), during these events huge complex IPSCs can be observed in target cells, resulting from the synchronized activity of many PVBCs (Hodapp et al., 2022). Among these targets are the major output neurons of the hippocampus, CA1 pyramidal neurons. Interestingly, simultaneous recordings from neurons in the deep layers of the entorhinal cortex that receive direct input from CA1 pyramids show that the greatest efficiency of this excitatory input is temporally tightly bound to SPW-R in the hippocampus (Rozov et al., 2020; Nasretdinov et al., 2023). Moreover, in the hippocampus and neocortex PVBCs are often reciprocally connected with excitatory neurons thereby creating microcircuits that can operate like micro-oscillators (Mann et al., 2005; Mann and Paulsen, 2005; Pastoll et al., 2013). Thus, PVBCs, providing highly effective and temporally precise inhibition, promote generation of rhythmic excitation. It is logical to assume that the amplitude and frequency of rhythmic excitation generated by PVBCs may remain not only within physiologically normal limits, but also reach the epileptic range if there is no system that counteracts PVBC-mediated hypersynchrony.

## Asynchronous perisomatic inhibition

In addition to PVBCs, perisomatic inhibition is provided by another type of interneuron, CCKBCs. These two types of interneuron can be found throughout areas of the hippocampus and neocortex (Whissell et al., 2015). Often, CCKBCs innervate the same targets as PVBCs. For example, in the hippocampal formation, granule cells in the *dentate gyrus* (Hefft and Jonas, 2005) and pyramidal neurons of the entire *stratum pyramidale* (Freund and Katona, 2007), as well as excitatory cells in the deep layer of the entorhinal cortex (Rozov et al., 2020; Nasretdinov et al., 2023) receive input from both PVBCs and CCKBCs. In some regions, CCKBCs and PVBCs control different types of excitatory cells (Fuchs et al., 2016; Witter et al., 2017). In many brain structures, CCKBCs and PVBCs receive excitatory input from the same neuronal populations (Freund and Katona, 2007; Freund, 2003). However, it should be noted that while PVBCs are largely involved in both feedback and feedforward inhibition, CCKBCs primarily mediate the latter (Nasretdinov et al., 2023; Espinoza et al., 2018; Liu et al., 2020). The high similarity of synaptic integration may suggest similar functions within these networks, but all available evidence suggests that the functions of the two types of perisomatic interneurons do not overlap. The difference between the network roles of PVBCs and CCKBCs most likely arises from the very different kinetics of GABA release during repetitive high-frequency firing characteristic of oscillatory activity. Unlike PVBCs, burst firing in CCKBCs causes asynchronous neurotransmitter release lasting tens to hundreds of milliseconds after the last action potential (AP) in the burst (Hefft and Jonas, 2005; Ali and Todorova, 2010). In fact, disruption of the synchrony of GABA release during prolonged high-frequency stimulation becomes evident after the 3rd or 5th AP in the train (Ali and Todorova, 2010; Daw et al., 2009). Moreover, CCKBCs express presynaptic cannabinoid receptors (CB1R), the activation of which shifts the ratio of synchronous and asynchronous release components toward the latter (Ali and Todorova, 2010). Thus, the involvement of CCKBCs in the generation of fast oscillations or in any precise transmission of information encoded in the frequency of presynaptic firing seems impossible. However, based on elegant *in vivo* juxtacellular recordings, it has been suggested that hippocampal CCKBCs may be involved in the generation and/or maintenance of theta oscillations (Klausberger et al., 2005). The authors showed that during theta oscillations, the maximum probability of CKKBC firing coincides with the peaks of the theta cycle. Klausberger's findings have been included into multiple *in silico* models of theta oscillations (Chatzikalymniou et al., 2021; Li et al., 2017). Interestingly, the average duration of asynchronous GABA release is about 50–150 ms, which matches the frequency of theta rhythms in rodents (4–10 Hz). It is likely that although CCKBCs may not be involved in the generation of fast rhythms, the asynchronous release of GABA at synapses formed by CCKBCs may play a vital role in the generation and/or maintenance of slow inhibitory waves underlying the theta rhythm. In a recent study, the involvement of CCKBCs in behaviorally driven oscillatory activity was assessed using *in vivo* optical calcium measurements (Dudok et al., 2021). They showed that in animals, CCKBCs are silent during theta activity associated with locomotion but active during the resting state. In addition, Dudok et al. demonstrated that the activity of CCKBCs significantly suppressed the firing of PVBCs and pyramidal neurons. Silencing of PVBCs by CCKBCs is in good agreement with the functional data provided by Karson et al (Karson et al., 2009), showing anatomical and electrophysiological evidence of direct inhibitory input from CCKBCs to PVBCs. They also show that high-frequency stimulation of this input results in robust asynchronous release of GABA. Data on the inhibitory control of PVBCs over CCKBCs are quite contradictory: Karson et al argue that there is no reciprocal communication, whereas Dudok et al demonstrate the existence of parvalbumin-positive GABAergic terminals on the soma and proximal dendrites of CCKBC. The latter may belong to bistratified parvalbumin-positive neurons. However, even if PVBCs innervate CCKBCs, the connectivity index is likely lower than that of CCKBCs-to-PVBCs because this type of connection was not found in paired recordings of identified neurons made by other groups. Another recent study showed that during optogenetic suppression of CCK-positive interneurons *in vivo*, CA1 pyramidal cell burst firing is enhanced, optogenetic suppression also caused phase shifts in CA1 pyramidal cell firing during theta but not gamma oscillations (Rangel Guerrero et al., 2024). Thus, all evidence to date suggests that, first, CCKBCs are involved in either the generation of slow oscillations or the suppression of rhythmogenesis by inhibiting PVBCs. Second, regardless of the experimental approach, juxtacellular recordings or measurements of intracellular calcium elevations as indicator of subthreshold activity, CCKBCs *in vivo* tend to fire bursts of APs that should result in asynchronous release and inhibition of target cells lasting longer than the detected firing activity.

## Antiepileptic mission of CCKBCs

Optogenetic manipulations of interneurons to control seizure activity in various animal models of epilepsy *in vivo* and *in vitro* fail to give a clear answer to the question of whether the stimulation of parvalbumin-positive interneurons actually prevents or favors seizure generation (Wykes et al., 2016; Krook-Magnuson et al., 2013; Shiri et al., 2015, 2016; Ewell et al., 2015; Yekhlef et al., 2015; Ledri et al., 2014; Ellender et al., 2014; Magloire et al., 2019). Since the major function of PVBCs is synchronization of network activity [as reviewed in Avoli and Curtis (2011); Jiruska et al. (2013)], it has been suggested that, under some circumstances, enhanced GABAergic signaling might actually trigger seizures.

CCKBCs are well adapted to counteract the hypersynchronizing proepileptic effects of PVBCs: (a) they are integrated into the same feedforward inhibitory circuits as PVBCs; (b) by inhibiting both PVBCs and pyramidal cells in reciprocally connected microcircuits, they can interrupt the maintenance of pathological oscillatory activity; and (c) seizure-associated repetitive high-frequency excitatory drive should result in asynchronous release of GABA from CCKBC terminals. Although the density of excitatory terminals converging on CCKBCs is lower than that of PVBCs, epileptiform activity recruits thousands of excitatory neurons, so this limitation should be easily overcome. Finally, neocortical CCK-positive interneurons, capable of generating asynchronous release in response to high-frequency stimulation, are integrated into feedforward inhibitory circuits between the hippocampus and a number of neocortical regions (Nasretdinov et al., 2023; Liu et al., 2020), the recruitment of these cells during epileptiform activity should prevent propagation of seizures from the hippocampus to distant parts of the brain (Figures 1B1, B2). Indeed, in line with this hypothesis, selective rapid loss of CCK-positive (but not PV-positive) GABAergic terminals has been shown to occur in a rodent models of temporal lobe epilepsy (Sun et al., 2014; Wyeth et al., 2010; Kang et al., 2021; Whitebirch et al., 2023). To induce temporal lobe epilepsy, different groups have used different strategies, including injections of kainate, pilocapine, or electrical stimulation. Loss of CCKBCs was observed regardless of the experimental approach used. Selective reduction of CCK-positive perisomatic terminals has not been confirmed in immunocytochemical studies of surgically excised temporal lobe samples from patients with epilepsy (Wittner and Maglóczky, 2017), however the functional contribution of CCKBCs to network regulation may be significantly reduced by activation of either presynaptic GABAB or CB1 receptors (Jappy et al., 2016).

## Role of the CB1R in CCK-positive interneurons as a stress sensor

An additional prominent and peculiar feature of nearly all CCK-positive interneurons including CCKBCs is the expression of presynaptically located cannabinoid receptors (CB1R). These receptors mediate an activity-dependent attenuation of GABA release which likely results in modulation of network activity. Indeed, several studies *in vivo* and *in vitro* show that the CB1R agonists THC (tetrahydrocannabinol) or WIN55212 reduce the power of theta, gamma and SPW-R oscillations (Holderith et al., 2011; Robbe et al., 2006; Robbe and Buzsáki, 2009; Soltesz et al., 2015). Although the above-mentioned studies clearly prove that CB1R serve as modulators of hippocampal rhythms, the underlying mechanisms and the specific role of CCK/CB1R-positive interneurons (CCK/CB1-INs) have not been elucidated.

Activation of presynaptic CB1Rs has a much stronger effect on the most synchronized synaptic events triggered by the first few APs in the train, while reduction of the late asynchronous component is less affected. Thus, endocannabinoids should preferentially reduce fast inhibition, which is important for signal transduction and network computation, without interfering with the antiepileptic properties of CCK/CB1-IN. The current view on the mechanism of endocannabinoid signaling suggests that an increase in postsynaptic calcium concentration triggers the synthesis and release of endocannabinoids with subsequent activation of presynaptic CB1Rs. The latter causes suppression of voltage-gated calcium channels, resulting in a temporary decrease in neurotransmitter release. However, there are other ways to increase endocannabinoid synthesis. It is known that the concentration of circulating 2-arachidonoyl glycerol increases in various brain regions including the hippocampus in response to glucocorticoid administration or as a result of acute restraint stress (Morena et al., 2016; Balsevich et al., 2017; Atsak et al., 2012a,b). In many brain regions CCK/CB1-INs, including CCKBCs, are involved in long-distance feedforward inhibition of both excitatory neurons and PVBCs, therefore stress-induced increases in endocannabinoid levels should lead to increased network synchronization and effectiveness of propagation of excitation. Indeed, in layer 5 of the entorhinal cortex, the impact of CCK/CB1R-IN inhibition on pyramidal cells is significantly reduced in animals exposed to acute inescapable stress, resulting in increased efficiency of SPW-R propagation from the hippocampus to the entorhinal cortex (Nasretdinov et al., 2023). The cellular and synaptic properties of deep entorhinal cortex CCK/CB1R-INs resemble those of hippocampal CCKBCs. Endocannabinoid modulation has also been shown for ventral hippocampus-driven inhibitory feedforward connections in the prefrontal cortex between CCK/CB1R-IN and layer 5 pyramidal cells (Li et al., 2017). Notably, connections from the ventral hippocampus to the prefrontal cortex regulate stress-sensitive higher brain functions such as cognition, emotion, and memory. In the hippocampus, acute stress modulation of CCKBC-mediated inhibition has not yet been studied in detail, however, given that hippocampal CCKBCs receive multiple excitatory inputs from long-range extra- and intrahippocampal projections (Bartos and Elgueta, 2012; Glickfeld and Scanziani, 2006), it is logical to hypothesize that CCKBC-mediated feedforward inhibition is sensitive to stress-induced endocannabinoid elevation. Nevertheless, strong contextual fear conditioning has been shown to induce biphasic expression of Npas4 in the hippocampus, leading to an increase in the number of CB1R-positive perisomatic terminals and resulting in a functional enhancement of CCKBC-mediated inhibition, which is hypothesized to contribute to the suppression of highly salient aversive experiences (Brito et al., 2024; Hartzell et al., 2018).

## Discussion

Parvalbumin-positive fast-spiking interneurons or PVBCs can ensure fast transmission of excitation between groups of different populations of neurons and different areas of the brain. These neurons have been described in almost all regions of the forebrain (Nahar et al., 2021; Druga et al., 2023). They have a number of important features: high-frequency firing of APs; high fidelity of GABA release and the ability to control spike generation in the target cell via synapses located on the soma and proximal dendrites (Milicevic et al., 2024). While PVBCs are inhibitory neurons, their main function is synchronization of excitatory cell firing and, therefore, promotion of excitation flow. However, hypersynchrony leads to the generation of pathological epileptiform activity (Ellender et al., 2014). Thus, to set the upper limit of synchronization, the brain needs another powerful inhibitory system, the main function of which should be desynchronization of neural networks. CCKBCs are the best candidate for this. Similarly to PVBCs, these interneurons are found throughout the forebrain and innervate the perisomatic compartment of target cells. In contrast to PVBCs, CCKBCs are irregular spiking interneurons and, most importantly, GABA release from the terminals of these cells exhibits a high level of desynchronization (Nasretdinov et al., 2023; Hefft and Jonas, 2005; Ali and Todorova, 2010). In addition, the density of excitatory terminals contacting CCKBCs is lower than that of PVBCs, which allows a mass involvement of CCKBCs in network activity when net excitation reaches pathological values (Freund and Katona, 2007). Second, in many brain structures, CCKBCs also innervate PVBCs (Karson et al., 2009), while the existence of the PVBC-to-CCKBC connections is still a matter of debate. Finally, CCKBCs are included in many long-range feedforward inhibitory circuits (Nasretdinov et al., 2023; Liu et al., 2020) that may help prevent seizure propagation from the focus of epilepsy to distant brain structures. One possible way to examine the antiepileptic role of CCKBCs would be the use of either optogenetic or chemogenetic silencing of these neurons *in vivo* in epileptic animal models.

In addition to the antiepileptic function CCKBCs may operate as a sensor of stress. Endocannabinoid suppression of GABA release from CCKBC terminals can rapidly disinhibit brain structures that are involved in the fast response to stress (Nasretdinov et al., 2023). Notably, stress dependent suppression of the inhibitory impact of CCKBCs strongly affects the synchronized phase of release and has a rather low effect on the asynchronous, antiepileptic component. Cotrarary enhancement of CCKBC-mediated inhibition might be involved in stress adaptation and the erasure of bad memories (Brito et al., 2024).

Thus, while PVBCs act as pacemakers, CCKBCs control their activities, acting as peacekeepers. This feature of CCKBCs makes them an interesting target for development of cell specific therapeutic approaches and for the development of novel antiepileptic and antidepressant drugs. For example, much attention is currently being paid to the medical use of CB1R agonists. Although synthetic agonists show some anticonvulsant effects in animal models, the results of clinical trials are less convincing. (Rosenberg et al., 2015). It is possible that treatment with CB1R antagonists, which leads to an increased influence of CCKBC on network activity, may yield better results. In the hippocampus CCKBCs belong to a rather small neuronal population that express ionotropic serotonin receptors (5HT3R) (Dale et al., 2016). Therefore, CCKBCs can be activated by selective 5HT3 agonists, which also opens up a possible direction for the development of new therapeutic approaches (Zhao et al., 2018).

As a final remark, we would like to suggest that, although PVBC and CCKBC may antagonize each other and appear to provide functionally different types of inhibition of target neurons, the similarity in their patterns of network integration makes these basket cells dialectically linked by the same global function—maintaining excitation flow and neuronal synchrony within the physiological range. In this regard, these two populations of interneurons can be used as an illustration of Hegel's first law of dialectics: “the unity and struggle of opposites,” formulated by the German philosopher more than 200 years ago.
